# Dr. John S. Failing

**Published:** 1906-07

**Authors:** 


Dr. John S. Failing, formerly of Grand Rapids, Mich., a graduate of the University of Buffalo, 1868, died recently at his home at Los Angeles, Cal.

